# Use of CLEANED to Assess the Productive, Environmental, and Economic Impact of Dairy Farms in the Peruvian Amazon

**DOI:** 10.3390/ani14223224

**Published:** 2024-11-10

**Authors:** Linda Oré, Gelver Romero, Maria H. Souza de Abreu, José Velarde-Guillén, Jacobo Arango, Juan Carlos Ku-Vera, Carlos Gómez

**Affiliations:** 1 Facultad de Zootecnia, Universidad Nacional Agraria La Molina, Av. La Molina s/n, La Molina, Lima 15024, Peru; oreramoslinda@gmail.com (L.O.); gromero@lamolina.edu.pe (G.R.); mhabreu@lamolina.edu.pe (M.H.S.d.A.); jguillen@lamolina.edu.pe (J.V.-G.); 2 Tropical Forages Program, International Center for Tropical Agriculture (CIAT), km 17, Palmira 763022, Valle del Cauca, Colombia; j.arango@cgiar.org; 3 Laboratory of Climate Change and Livestock Production, Department of Animal Nutrition, Faculty of Veterinary Medicine and Animal Science, University of Yucatan, Mérida 97315, Yucatán, Mexico; kvera@correo.uady.mx

**Keywords:** sustainability, tropical, dairy farm, greenhouse gas, grazing, environment, animal production

## Abstract

Dairy farms in developing countries are key to fighting hunger and poverty. However, their environmental and economic impact in the tropical region of Peru has been scarcely studied. The CLEANED tool, developed by the Alliance of Bioversity International and the International Center for Tropical Agriculture, allows us to assess the productive situation and the environmental and economic impact of different dairy systems. The objective of the study was to use the CLEANED tool to evaluate the production, environmental, and economic impacts of extensive and semi-intensive dairy systems in the tropical region of Peru. Data from twelve dairy farms (six extensive and six semi-intensive systems), in two seasons (rainy and dry) were recorded using semi-structured surveys. The required area (ha/year), production (L milk/ha/year), environmental impact (water use in m^3^/product), and methane (CH_4_) and nitrous oxide (N_2_O) emissions in kg CO_2_eq/kg fat- and protein-corrected milk (FPCM) were estimated, and the economic analysis was performed for each livestock system. Results showed that the required area was of 28.9 and 32.6 ha, milk yield was 382 and 1254 L/ha/year, water use was 0.59 and 0.29 m^3^/kg FPCM, CH_4_ emissions were 1.7 and 1.0 kg CO_2_eq/kg FPCM, and N_2_O emissions were 0.22 and 0.17 kg CO_2_eq/kg FPCM, for the extensive and semi-intensive systems, respectively. The study revealed significant positive differences in semi-intensive livestock systems, including increased productivity, the reduced intensity of CH_4_ and N_2_O emissions, and greater profitability.

## Simple Summary

The CLEANED tool, developed by the Alliance of Bioversity International and the International Center for Tropical Agriculture, allows for a better understanding of the productive status and the environmental and economic impacts of different dairy systems. This work compares the following two dairy systems in the Peruvian Amazon: extensive (grazing) and semi-intensive (grazing plus supplementation). Results showed that farmers may likely need a smaller area than what they use at present (grazing area). Milk yield was 382 and 1254 L/ha/year, water use was 0.59 and 0.29 m^3^/kg of fat- and protein-corrected milk (FPCM), methane (CH_4_) emissions were 1.7 and 1.0 kg CO_2_eq/kg FPCM and N_2_O emissions were 0.22 and 0.17 kg CO_2_eq/kg FPCM, for the extensive and semi-intensive systems, respectively. The assessment of the status of dairy farms under tropical conditions allows the development of strategies to mitigate greenhouse gas emissions, while increasing revenue by increasing the milk yield or decreasing the feeding costs.

## 1. Introduction

Global trends indicate that between 2030 and 2050, consumer demand for animal products (meat, milk, eggs, fish) will increase by up to eight times compared to its current value [1]. This means that there is a need to increase the production of food of animal origin, especially in developing countries. However, livestock production has an important environmental impact, especially with regard to greenhouse gas emissions, deforestation, water pollution, among others [2].

In the Peruvian Amazon, agriculture is a strategic activity, because it allows access to capital and the products it generates [3,4], but with a direct environmental impact. MINAM [5] estimates that from 2001 to 2019 the Peruvian Amazon lost an average of 130,500 hectares per year, with agriculture responsible for between 49 and 54% of total deforestation.

On the other hand, livestock farming in the Peruvian Amazon plays an important function in economic, social, and environmental cohesion [6]. In addition, dairy farms are recognized for their contribution to the food security of rural families and for generating work [7,8]. The Peruvian Amazon produces 8.5% of the national milk production [9].

In the Peruvian Amazon there are no specialized dairy farms; all are dual-purpose, used for both milk production and the sale of calves or fattening. In the region there are two main feeding strategies, extensive (grazing) and semi-intensive (grazing plus supplement), with Gyr × Holstein or Gyr × Brown Swiss dairy cows. Moreover, there are factors that limit dairy production in the region, such as the low quantity and quality of feed, mainly in the dry season [10,11], and animal health and other problems that are exacerbated due to climate change [12]. In addition, at present, there is no information available on the environmental and economic impact of dairy production systems in the Peruvian Amazon.

To assess the environmental and economic impact of the different dairy systems in the tropics, the Comprehensive Livestock Environmental Assessment for Improved Nutrition, a Secured Environmental and Sustainable Development along Livestock Value Chain (CLEANED) tool was developed by the International Center for Tropical Agriculture (CIAT) in partnership with the Consultative Group on International Agricultural Research (CGIAR), International Livestock Research Institute (ILRI), and Biodiversity International in 2013 and 2014, as an easily adaptable and transferable indicator framework that takes into account the entire value chain. The tool has been updated, generating the new versions CLEANED (R); CLEANED X-Version 2.0.1; and CLEANED X-Version 3.0.1 [13]. The tool was used in different countries in Africa and Central America, carrying out assessments in groups of small farmers, providing them with information on their productive and environmental status and suggesting possible alternatives for improving animal production [14,15].

The CLEANED tool calculates the economic, productive, and environmental impact of animal production. The above are calculated using land requirements, productivity (milk and meat yield), economics (costs, milk and meat price), soil impacts (e.g., erosion, N balance), greenhouse gas emissions (GHGe), and water impacts.

The objective of this study was to evaluate the productive, environmental, and economic impacts of extensive and semi-intensive dairy farming systems in the tropical region of Peru, using the CLEANED tool.

## 2. Material and Methods

The study was conducted in accordance with the guidelines and regulations of the Institutional Committee for the Care and Use of Animals (IACUC) of the National Agrarian University La Molina (TR.N°0185-2016-CU-UNALM).

### 2.1. Area of Study

**Figure 1:**
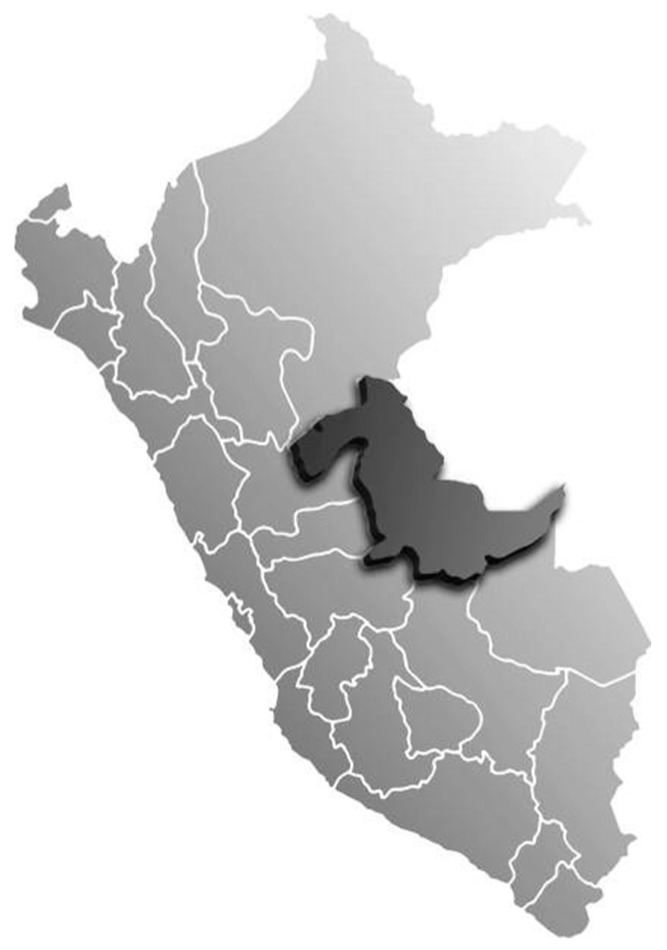
Ucayali region, Peru.

Data from 12 dairy farms from the Ucayali region in Peru (9°58′ S and 73°11′ W) were used (Figure 1). The Ucayali region has an altitude between 154 and 287 m.a.s.l. The average temperature in the region is 28 °C and annual precipitation is 1566 mm.

### 2.2. Dairy Farms

Dairy farms in the Peruvian Amazon were characterized by having dairy cows of two main genotypes as follows: Gyr × Holstein and Gyr × Brown Swiss. The distribution of the dairy cows’ genotypes is heterogenous in the region. The main management of the farms follows a dual-purpose system, by which the offspring is maintained with the cow until it is weaning. The weaning period in these farms is 6 months. Animals are hand-milked once daily. Farmers generally consider the calf growth rate more important than the milk yield. Consequently, suckling is commonly allowed without restriction.

The most frequently used feeding strategy is grazing. The types of grass used for grazing are the following: *Brachiaria decumbens*, *B. dictioneura*, *B. brizantha*, *Hyparrhenia rufa*, and *Andropogon gayanus*, while *Pennisetum purpureum* × *Pennisetum typhoides* is used for cut-and-carry.

### 2.3. Source of Information

The 12 farms sampled were selected from a list of 45 preselected milk producers through field surveys, which met the following predetermined characteristics: grazing without supplementation, and grazing with supplementation. Dairy farms were grouped into the following two groups (six farms each): (1) extensive or pastoral production system, which is based on grazing without supplementation, and (2) semi-intensive system, in which the feeding strategy is based on grazing or cut-and-carry or both, with supplementation (balanced feed and/or brewery waste). Nutritional values are presented in Table 1.

In each system, information was collected during the rainy season and the dry season of the year 2022. The interviews were conducted by personnel trained in the structure of the questionnaire and in the operation of the CLEANED tool. The interviews were answered by the manager of each farm. Surveys were carried out based on the main characteristics of the farms, including total area, number of hectares of pasture, number of animals on the farm, herd categories, quantity of kg of milk/cow/day, sale price of products generated on the farm, farm expenses and income, pasture management, and infrastructure. Data of the surveys were entered into the CLEANED tool. In addition, forage, supplement, and milk samples were collected. The milk, grass, and supplement samples were analyzed in the Food Nutritional Evaluation Laboratory of the National Agrarian University La Molina. Milk was analyzed for milk grass and milk protein content. Meanwhile, the feed samples were analyzed for dry matter, crude protein, neutral detergent fiber, acid detergent fiber, ash, and ether extract.

### 2.4. CLEANED Tool

For this work, the CLEANED X Version 3.0.1 was used. CLEANED modeled the response variables, such as the surface area required to carry out the livestock activity (pasture area ha/year); production kg FPCM/ha/year and kg meat/ha/year; environmental impacts, through the use of water (m^3^/kg product), methane (CH_4_), and nitrous oxide (N_2_O) emissions, expressed in t of CO_2_eq/ha/year and kg CO_2_eq/kg FPCM; and changes in carbon storage (t CO_2_eq/ha/year) by mineralization in the soil and carbon sequestration by above-ground biomass (trees). The information is processed on the desktop interface using the Run program icon or by proceeding to R software calculations.

The information obtained on the characteristics of the evaluated systems (Table 1), the productive data, and nutritional value of the pasture and milk of the systems, both in the dry and rainy seasons, was entered into the spreadsheets of the CLEANED X Version 3.0.1 tool. Likewise, for the estimation of GHG emissions, enteric fermentation, the use of manure, and soil management were considered as emission sources, as per the principles established by Tier 1 (IPCC, [16]). Land requirement is calculated using the data of the number of animals, dry matter intake, and forage yield.

The nutritional value of the feed was taken from the analyses carried out and used in the calculations of the CLEANED tool. The total feeds were calculated for a dairy cow and presented using the live weight (LW). The total feed supply was compared with the needs of the cattle. The requirements were as follows: a daily intake of 2.5% per kg of live weight of dry matter (DM) (LW); for crude protein (CP), 6.27 g/kg per metabolic weight for maintenance (507 g/LW), and 92.6 g of CP/L of the milk produced. For metabolizable energy, 0.598 MJ/kg metabolic weight was used for maintenance and 5.65 MJ/L for milk production.

### 2.5. Economic Evaluation

The economic analysis was determined from the annual income and expenses, to finally obtain the profit. Total income was obtained from the sum of milk sales/farm/year and animal sales by category. Expenses were obtained from fixed costs (land, machinery and equipment, vehicles, facilities and civil works, pastures, and animals) and operational costs (labor, supplementary feeding, materials and supplies, third-party services, maintenance and repair of equipment, and depreciation). The profit per year was divided by the total number of animals in the herd, obtaining the profitability (%) of the extensive and semi-intensive systems.

### 2.6. Statistical Analysis

Statistical analysis was performed using SAS software (version 9.4., SAS Inst., Cary, NC, USA). Normal distribution was tested using Shapiro–Wilk tests. Means of quantitative data that followed a normal distribution were compared using Student’s *t* test (parametric tests), using both the confidence interval estimation analysis and t-score probability hypothesis testing method for the two independent sample groups.

## 3. Results

### 3.1. Dairy Farm Characteristics

Semi-intensive systems had 86% more animals, 111% more cows in production, and 32% higher milk production/cow/day compared to extensive systems (Table 1). The categories of the evaluated herds were distributed as follows: cows in production, 27.8 and 28.4%; dry cow land, 34.8 and 14.0%; young bulls, 19.6 and 21.4%; bulls, 2.1 and 6.0%; and rearing, 15.7 and 30.3%, in the extensive and semi-intensive systems, respectively. The total area and grazing area (ha) did not show any difference (*p* > 0.05). Likewise, the nutritional values of milk in the evaluated systems do not show any difference (*p* > 0.05).

### 3.2. Required Area

**Figure 2:**
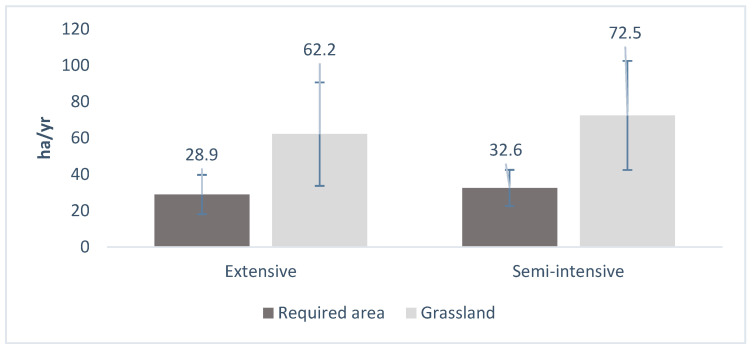
Required area in comparison with grassland area in dairy farms under extensive and semi-intensive systems in the Peruvian Amazon. Despite farm management, total area for grazing and required area presented no differences (*p* = 0.559) between systems of production.

The tool showed that the optimal area of pasture (Figure 2), under the conditions mentioned above for feeding animals, does not show any difference (*p* = 0.559). Semi-intensive systems require only 44% of the area currently used for pasture, while the same pattern is observed in extensive systems, where only 46% of the surface area in use is required. This reveals an under-exploitation of resources in both systems (semi-intensive and extensive).

### 3.3. Milk and Meat Production

Table 2 shows milk production by system, with a significant difference (*p* < 0.01), with the semi-intensive system being considerably higher (3.3 times in kg FPCM/ha/year) than the extensive system. As for meat production, there was no significant difference (*p* > 0.05), though the semi-intensive system showed an increase of 1.6 times more meat/ha compared to the extensive system.

### 3.4. Environmental Impacts

Table 3 shows the significant differences (*p* < 0.01) in the water use, with the semi-intensive system reducing the use of water per kg FPCM and meat and protein by 50.8, 12.5, and 45.7%, respectively, compared to the extensive system. Soil erosion did not show any significant differences (*p* > 0.05). Regarding carbon storage, a similar behavior is observed (*p* > 0.05), with greater carbon storage in semi-intensive (0.18 Mg C./ha/year) than in extensive systems (0.14 Mg C./ha/year).

Methane emissions were 379 and 309 g/cow/day in the extensive and semi-intensive systems, respectively. The intensity of methane emissions in the semi-intensive system was reduced by 41% compared to the extensive systems (Table 3). In the same line, N_2_O emissions decreased by 23% in the semi-intensive system.

GHG emissions per area showed significant differences (*p* < 0.01; Table 4). Semi-intensive systems emitted 98% more GHG per ha/yr. Carbon sequestration converted into Mg CO_2_eq/ha/year was used to calculate net GHG emissions, showing no difference (*p* = 0.2025).

### 3.5. Economic Analysis

The income and total annual expenses showed significant differences (*p* < 0.05; Table 5), with the semi-intensive system being 5.3 and 5.1 times higher, respectively. The income in the semi-intensive system is distributed as follows: milk sales, 92.6%; animals, 4.5%; and crops, 2.9%. As for the extensive system, 89.1% came from milk sales, 8.4% from animals, and 2.5% from crops. The difference in annual profitability was not statistically significant (*p* > 0.05). The sale price per liter of milk was $0.41 dollars.

## 4. Discussion

### 4.1. Required Area

In dairy farming, pasture availability plays a crucial role in efficiency and sustainability. Previous research reports that extensive systems require a larger surface area than semi-intensive systems to produce the same amount of product [17]. In this work, results showed an animal load of 0.36 and 0.74 AU/ha for extensive and semi-intensive systems, respectively. In the same line, Murgueitio et al. [18] report an animal load of 0.59 animals/ha under similar conditions in the tropics. According to Lopes et al. [19], the carrying capacity of *Brachiaria brizantha* cv. ranges between 1.5 and 2.4 AU/ha. The above shows that there is undergrazing of the grassland, which reveals an opportunity for its optimization.

### 4.2. Milk and Meat Production

In general, the semi-intensive system is more efficient in the production of milk and meat than the extensive system [20]. The above is in line with the observations in this work, in which the highest milk and meat production was found in the semi-intensive system, mainly due to the greater number of lactating cows, greater amount of milk/cow/day, as well as greater number of calves for meat production. Bashir and El Zubeir [21] and Rojo-Rubio et al. [4] reported a similar productive behavior in milk in extensive and semi-intensive systems in Baggara cattle and dual-purpose cattle in the Mexican tropics. Murgueitio et al. [18] reported values like those found in this study (89.7 and 19.9 kg of milk/ha/yr and kg of meat/ha/yr, respectively). However, it is important to highlight that the extensive system presents certain advantages, such as greater diversity and less use of external resources [22,23]. Supplementation strategies for cattle grazing in tropical pastures usually aim to guarantee the supply of fermentable nitrogen (N-NH_3_) for the rumen microbial population as well as the minerals (phosphorus, sulfur, magnesium) necessary for microbial growth, altogether leading to a greater synthesis of microbial protein in the rumen [24] and, eventually, to a higher productive performance.

### 4.3. Environmental Impacts

The semi-intensive system presented greater efficiency in the use of water per product due to the use of external inputs added to management practices. Mekonnen and Hoekstra [25] reported that beef production requires an average of 15,415 L of water per kg of meat, while milk production requires an average of 6878 L of water per kg of milk. These values are significantly higher than those found in this study. Efficient water use is directly related to the type of forage crop used in the Amazon. In this regard, studies carried out on livestock systems in the tropics of Nicaragua [26] and Tanzania [27] showed similar results.

Soil erosion observed in this study was influenced by certain environmental factors [10] such as grazing, animal load, and the topography of the pastures, especially in areas with a steep slope and without vegetation cover. Areas with steep slopes are more prone to erosion [28] and may cause greater loss of soil, and therefore loss of soil organic carbon too [29].

Regarding soil carbon storage, a greater carbon sequestration was observed in the semi-intensive system compared to the extensive system. The factors that could have influenced this are the vegetation cover and grazing management, since the semi-intensive systems were better managed, mainly due to a greater presence of trees in their pastures [30,31]. Dondini et al. [32] estimated that the carbon sequestration potential in pastoral systems ranges between 0.18 and 0.41 tons of C/ha/yr in all the different regions of Sub-Saharan Africa, South Asia, and Latin America, values similar to those found in this study. In general, tropical regions have low carbon reserves [32], which have great potential for carbon storage in the soil.

### 4.4. Greenhouse Gas Emissions

The results of this study showed higher GHG emissions in the semi-intensive systems since this system has a higher animal load and higher productivity [33,34]. Regarding the intensity of GHG emissions, the semi-intensive system presented lower CH_4_ and N_2_O emissions per kg of FPCM. Several investigations show that enteric fermentation is the main source of methane emissions in livestock systems [35,36]. Likewise, the intensity is correlated with the quality of feed supplied to the animal [37,38]. In the case of the semi-intensive systems of the surveyed farms, lower GHG emissions were reported per kg CO_2_eq/kg FPCM, probably due to better diet quality. These values are similar to the values reported by Ruiz-Llontop et al. [39] for silvopastoral dairy systems in the northern Amazon of Peru. Berton et al. [40] reported emissions of 1.0 ± 0.3 kg CO_2_eq per kg of FPCM in livestock systems. Furthermore, it has been widely documented that extensive livestock farming in the tropics, based only on naturalized grasses, has negative implications in terms of the intensity of enteric methane emissions, while animals with a better energy–protein balance in their diet (supplementation) produce less of this gas [41,42]. Kliem et al. [43] reported a reduction in GHG emissions due to supplementation with agro-industrial by-products, which has occurred in this study in semi-intensive systems, which received supplementation with brewery waste.

Average net GHG emissions on livestock farms associated with the dairy production system were positive. The estimated net GHG emissions ranged from −1.15 to 1.24 Mg CO_2_eq/ha/yr in the extensive system, while the semi-intensive system ranged from −0.52 to 2.72. Our average estimate of net GHG emissions per ha is within the range of some of the values reported for grasslands in temperate climates. However, studies of net GHG under these conditions are limited. For example, in Spain [44] and Ireland, Fornara et al. [45] estimated net GHG emissions per ha for dairy production to be between 4.8 and 6.8 Mg CO_2_eq/ha/yr, using previous IPCC [16] reports.

### 4.5. Economic Analysis

The results obtained in this study are consistent with the existing literature, which suggests that production systems under a semi-intensive system generally require a higher initial investment [46,47]. However, in the long term, they can generate higher income and benefits [48]. In this case, the semi-intensive system presents an annual profitability of 19.14%, while the extensive system reaches 14.01%. Murgueitio et al. [49] mentioned that semi-intensive livestock systems in Latin America tend to be more profitable than silvopastoral and pastoral (extensive) systems. The increase in the utility of the semi-intensive system is due to its high ratio of number of animals in production, liter of milk produced (8.6 L/cow/day), and sale of animals, among other benefits. It is important to highlight that, in addition to the economic aspects, the choice of the appropriate production system must consider other factors such as climatic conditions, the availability of natural resources, accessibility, and the preferences of producers.

## 5. Conclusions

The semi-intensive system presented significant positive differences in production, environmental, and economic impacts, due to increased productivity and greater efficiency in the use of water and outputs (production of milk, meat, and proteins), reducing the intensity of CH_4_ and N_2_O emissions and presenting a higher annual profitability.

## Figures and Tables

**Table 1:** Characteristics of dairy farms under extensive and semi-extensive systems in the Ucayali region, Peru.

Item	Unit	Extensive System	Semi-Intensive System	*p* ^1^
Animals	Head	29 ± 8 ^2^	54 ± 25	0.0378
Dairy cows	Head	9 ± 4	19 ± 6	0.0116
Total area	Ha	87.8 ± 60.7	91.7 ± 42.5	0.9017
Grassland	Ha	62.2 ± 28.5	72.5 ± 30.0	0.5538
Milk yield	L/cow/d	6.5 ± 0.9	8.6 ± 0.5	0.0005
Milk fat	%	2.7 ± 0.5	3.1 ± 0.2	0.2678
Milk protein	%	3.5 ± 0.1	3.4 ± 0.1	0.4611
Ingredients	Unit	*Brachiaria brizantha*	Brewery waste	Supplement *
Dry matter	%	31.14	27.46	93.18
Crude protein	%	2.33	8.15	12.19
Digestibility of the dry matter	%	53.13	36.725	59.7
Neutral detergent fiber	%	20.97	14.72	31.29

* Mix of brewery byproduct, rice powder, wheat byproduct, soy, and minerals. ^1^ Values differ significantly at *p* < 0.05; ^2^ Mean values.

**Table 2:** Milk and meat production of dairy farms under extensive and semi-extensive systems in the Ucayali region, Peru.

Item	Unit	Extensive System		Semi-Intensive System		*p* Value
		Average	SD	Average	SD	
Milk yield	kg FPCM */yr	27,737	15,978	71,645	20,218	0.001
	kg FPCM/ha/yr ^1^	382	167	1259	814	0.092
Meat production	kg/yr	697	297	1184	563	0.090
	kg/ha/yr	10.3	5.3	16.4	2.4	0.878

* FPCM: Fat- and protein-corrected milk; ^1^ Area = grassland.

**Table 3:** Environmental impact of dairy farms under extensive and semi-extensive systems in the Ucayali region, Peru.

Item	Unit	Extensive System		Semi-Intensive System		*p* Value
		Average	SD *	Average	SD	
Water use	m^3^/kg of FPCM	0.59	0.17	0.29	0.07	0.007
	m^3^/kg of meat	22.29	8.25	19.51	7.85	0.600
	m^3^/kg of protein	14.36	2.33	7.80	1.70	0.001
Soil erosion	t/ha/yr	1.53	0.43	1.85	1.12	0.560
Carbon stock	Mg C/ha/yr	0.14	0.04	0.18	0.9	0.350
Methane	kg CO_2_eq/kg FPCM	1.70	0.51	1.00	0.17	0.010
Nitrous oxide	kg CO_2_eq/kg FPCM	0.22	0.05	0.17	0.03	0.032
GHG emissions	kg CO_2_eq/kg FPCM	1.91	0.49	1.18	0.19	0.007
	kg CO_2_eq/kg meat	72.43	24.36	84.03	33.71	0.510
	kg CO_2_eq/kg protein	46.94	6.56	31.75	3.74	0.001

* SD: Standard deviation; FPCM: Fat- and protein-corrected milk; GHG: Greenhouse gas.

**Table 4:** Net greenhouse gas emissions (Mg CO_2_eq/ha/yr) of dairy farms under extensive and semi-extensive systems in the Ucayali region, Peru.

Item	Extensive System		Semi-Intensive System		*p* Value
	Average	SD *	Average	SD	
GHG emissions	1.32	0.74	2.62	0.42	0.005
Carbon sequestration	0.90	0.96	0.66	0.32	0.580
Net emission	0.64	1.01	1.57	1.19	0.203

* SD: Standard deviation; GHG: Greenhouse gas.

**Table 5:** Economic analysis, in US dollars, of dairy farms under extensive and semi-extensive systems in the Ucayali region, Peru.

Item	Extensive System	Semi-Intensive System	*p* Value
Total inversion	11,002	116,475	0.052
Annual income	4396	23,340	0.008
Annual expenses	3786	19,525	0.009
Gross profit per year	604	3815	0.016
Gross profit per month	50	318	0.016
Annual profitability	14	19	0.213

## Data Availability

The data are available with a justified cause. The model is available at https://alliancebioversityciat.org/es/tools-innovations/cleaned (accessed on 1 July 2024).
